# Changes in clinical practice patterns and outcomes of hyponatremia after implementation of electronic alerts for electrolyte disturbances

**DOI:** 10.1093/ckj/sfag383

**Published:** 2026-10-30

**Authors:** Miho Murashima, Minamo Ono, Masashi Mizuno, Takayuki Hamano

**Affiliations:** Department of Nephrology, Nagoya City University, Graduate School of Medical Sciences, Aichi, Japan; Division of Nephrology, Department of Internal Medicine, Kindai University Faculty of Medicine, Osaka, Japan; Department of Nephrology, Nagoya City University East Medical Center, Aichi, Japan; Department of Nephrology, Nagoya City University, Graduate School of Medical Sciences, Aichi, Japan; Department of Nephrology, Nagoya City University, Graduate School of Medical Sciences, Aichi, Japan; Department of Nephrology, The University of Osaka Graduate School of Medicine, Osaka, Japan

**Keywords:** electrolyte disturbances, electronic alerts, hyponatremia

## Abstract

**Background:**

Electrolyte disturbances are common but are frequently overlooked. We investigated whether clinical practice and outcomes changed after implementation of electronic alerts (e-alerts) for electrolyte disturbances.

**Methods:**

This single-center, retrospective cohort study was conducted at a university hospital that implemented the e-alert system “Electrolyte Busters” in May 2021. E-alert criteria were serum sodium <125 or >160 mEq/L, potassium <2.5 or >6.0 mEq/L, corrected calcium <7.5 or >11.5 mg/dL, and magnesium <1.0 or >4.0 mg/dL. Outcomes among patients with hyponatremia were compared between the years before and after e-alert implementation. Urinary chemistry measurements, treatment and overcorrection were analyzed using logistic regression. Referral and fall rates were analyzed using incidence rate ratios (IRRs) and negative binomial regression, respectively. Length of stay (LOS) was compared using the Mann–Whitney U test.

**Results:**

Data from 36 379 pre-intervention and 37 395 post-intervention patients were analyzed. Median age was 62 years, and 48% were male. After e-alert implementation, nephrology referrals for hyponatremia increased from 1.1 to 2.3 per month [IRR (95% confidence interval) 2.07 (1.04–4.36)]. Urinary chemistry measurements increased [odds ratio (OR) (95% confidence interval) 1.62 (1.08–2.44)], whereas untreated cases decreased [OR 0.31 (0.19–0.53)]. Median (interquartile range) LOS decreased from 27 (15–47) to 20 (12–36) days (*P* = .01). Hyponatremia overcorrection [OR 0.64 (0.36–1.14)] and fall rates [IRR 0.82 (0.36–1.69)] showed downward trends.

**Conclusion:**

E-alerts were significantly associated with improved diagnostic evaluation and therapeutic interventions, and shorter LOS among patients with hyponatremia.

## Graphical Abstract

**Graphical Abstract:**
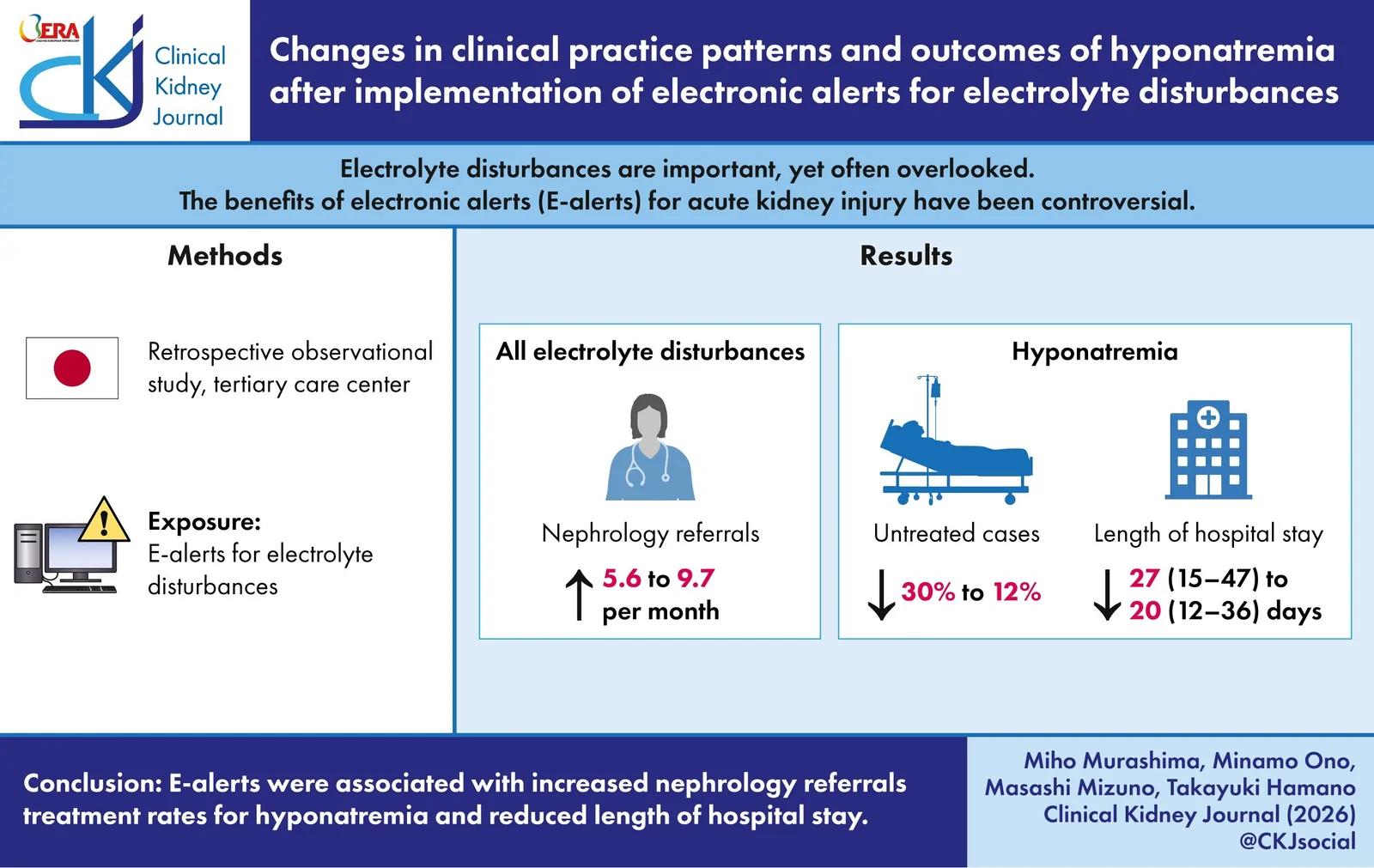

KEY LEARNING POINTS **What was known:**

- Electrolyte disturbances are common, but are often overlooked or untreated.

**This study adds:**

- E-alerts for electrolyte disturbances were associated with an increase in nephrology referrals.
- E-alerts for hyponatremia were associated with an increase in urinary sodium and osmolarity measurements and treatments.
- E-alerts for hyponatremia were associated with shorter length of stay.

**Potential impact:**

- E-alerts for electrolyte disturbances may improve the recognition and outcomes of hyponatremia.

## INTRODUCTION

Electrolyte disturbances are common in clinical practice but are often overlooked or mismanaged. Only about one-third of patients with malignancy-associated hyponatremia underwent urine sodium and osmolality measurements, although these tests are fundamental for determining the underlying cause [1].

Electronic alerts (e-alerts) were developed to improve recognition and outcomes of medical conditions. E-alerts for acute kidney injury (AKI) have been extensively studied. However, their efficacy remains controversial. An observational study reported less AKI progression to higher stages and greater likelihood of recovery [2], whereas a randomized controlled study found increased nephrology referrals without difference in the progression of AKI or in-hospital mortality [3]. In a recent meta-analysis, e-alerts for AKI were associated with less AKI progression but not with mortality [4]. Targeting a specific population might be more appropriate [5].

Despite the uncertain efficacy of e-alerts for AKI, we hypothesized that e-alerts may be particularly beneficial for electrolyte disturbances, because electrolyte disturbances are frequently overlooked, and are treatable, whereas disease-specific therapies are limited for many causes of AKI. Moreover, treating hyponatremia may improve bone turnover [6], neurocognitive function [7] and gait disturbance [8], and has been associated with lower mortality [9].

Nagoya City University Hospital implemented e-alerts for electrolyte disturbances in May 2021. Among multiple electrolyte disturbances, we focused on hyponatremia as treatment has been associated with improved outcomes. This study aimed to examine whether implementation of e-alerts was associated with increased nephrology referrals, and changes in clinical practice and outcomes among patients with hyponatremia.

## MATERIALS AND METHODS

### Study design and ethical considerations

This is a single-center, retrospective observational study. The study protocol and waiver of consent were approved by the Nagoya City University Graduate School of Medical Sciences and Nagoya City University Hospital Institutional Review Board (Approval No. 60-24-0062), and the study was conducted in accordance with the Helsinki Declaration.

### Settings and patients

E-alerts for electrolyte disturbances were implemented at Nagoya City University Hospital in May 2021. Fig. 1 shows an example of the e-alert screen and thresholds for e-alerts. The criteria for e-alerts were serum sodium <125 or >160 mEq/L, potassium <2.5 or >6.0 mEq/L, corrected calcium <7.5 or >11.5 mg/dL, and magnesium <1.0 or >4.0 mg/dL. Based on preliminary investigation, thresholds were set below the 1st percentile or above the 99th percentile for each electrolyte value at Nagoya City University Hospital (Supplementary data, Fig. S1), in order to minimize alert fatigue and to avoid excessive nephrology consultations. When laboratory results met the alert criteria, a message appears when the ordering physicians access the patient’s electronic medical record: “Abnormal values for electrolytes were detected. Please consult nephrology if necessary” (Fig. 1). The e-alerts appear repeatedly until acknowledged. No decision support, such as recommendations for urine electrolyte measurements, is provided. We enrolled those who underwent electrolyte measurements during the year before (from May 2020 to April 2021) or after (from May 2021 to April 2022) e-alert implementation. For analyses of those with an alert for hyponatremia, we excluded patients presenting with cardiopulmonary arrest or terminal illness (defined as expected death within a month, such as advanced cancer), as these patients were unlikely to benefit from nephrology referrals. Patients transferred to other facilities from the emergency department, pediatric patients (aged <18 years) and those on dialysis were also excluded (all patients on dialysis were routinely referred to nephrologists at our facility).

**Figure 1:**
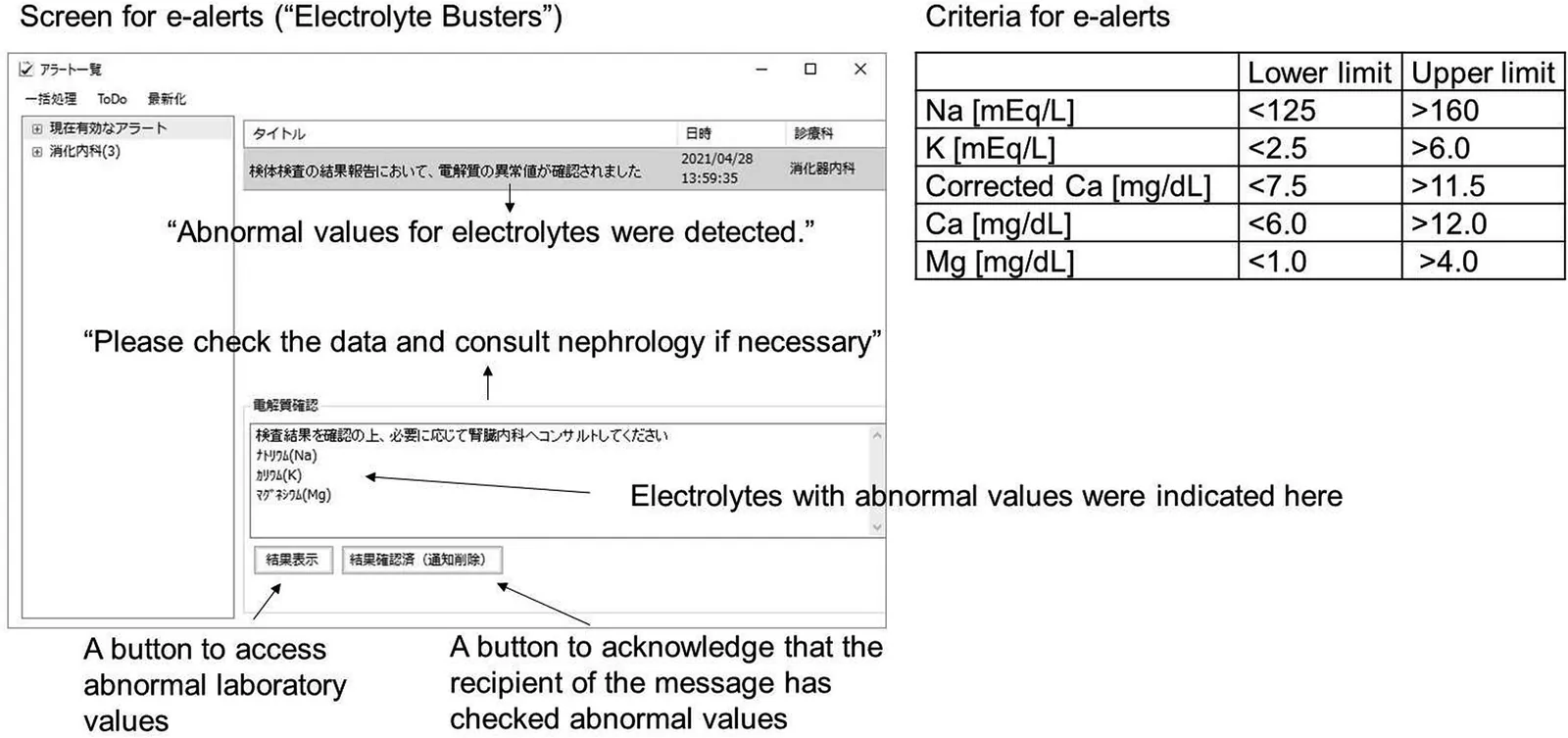
An e-alert screen and the criteria for alerts English translation is described next to Japanese text.

### Outcomes of interest

Predefined outcomes were compared before and after e-alert implementation. These included the number of nephrology referrals for electrolyte disturbances, and among patients with an alert for hyponatremia, urinary sodium concentration and osmolarity measurements, treatments for hyponatremia, length of stay (LOS), falls during hospitalization and overcorrections of hyponatremia. Analyses of nephrology referrals, urine chemistry measurements and treatments included both inpatients and outpatients. On the other hand, analyses of LOS, falls and overcorrection included only inpatients. All the consultation text during the study period was automatically extracted from the electronic health record and manually reviewed by two authors (M.O. and M.Murashima). The search terms were “hyponatremia,” “hypernatremia,” “hypokalemia,” “hyperkalemia,” “hypocalcemia,” “hypercalcemia,” “hypomagnesemia” and “hypermagnesemia.” Consultations containing these terms were classified as related to electrolyte disturbances. One patient could contribute more than one referral, if referred for multiple electrolyte disturbances. On the other hand, for analyses focused on hyponatremia (urine chemistry measurements, treatment, LOS, falls and overcorrection), only the most severe event, defined by the lowest serum sodium concentration, was evaluated for patients with multiple episodes of hyponatremia. Treatment for hyponatremia was identified from documentation in the medical chart. Cases were classified as treated if a management change was documented in response to hyponatremia, such as modification of intravenous fluid. Treatments were categorized as follows, intravenous NaCl (any intravenous fluid containing NaCl, including normal saline or addition of 10% NaCl to maintenance fluid), hypertonic NaCl (intravenous fluid containing Na >155 mEq/L), oral NaCl, cessation or reduction of loop or thiazide diuretics, tolvaptan [including tolvaptan use for syndrome of inappropriate antidiuresis (SIAD) or for heart failure] and loop diuretics (including use for heart failure or for SIAD with oral NaCl). The overcorrection of hyponatremia was defined as an increase in sodium concentration >10 mEq/L during the first 24 h and >8 mEq/L/day thereafter, according to the guideline from the European Society of Endocrinology, European Society of Intensive Care Medicine and European Renal Association–European Dialysis and Transplant Association [10].

### Statistical analyses

Data were presented as number (%), mean (standard deviation) or median (interquartile range). We used standardized differences rather than *P*-values as the sample size was large. *P*-values depend on sample size—when a sample size is large, clinically meaningless small differences between groups can translate into significant *P*-values (*P* < .05). On the other hand, standardized differences do not depend on sample size, and standardized differences of more than 10% are generally considered meaningful differences between groups [11].

The incidence rate ratio (IRR) for nephrology referrals for electrolyte disturbances before and after e-alert implementation was estimated using the immediate IRR calculator (iri command in Stata). The incidence rate was calculated as the number of nephrology referrals per number of electrolyte measurements during each observation period. Associations of e-alert implementation with urine chemistry measurements, treatment for hyponatremia and overcorrection of hyponatremia were evaluated using logistic regression. LOS before and after e-alert implementation was compared using the Mann–Whitney U test. The IRR for falls during hospitalizations was estimated using negative binomial regression. The incidence rate for falls was calculated as the number of falls per admission. The statistical analyses were performed using STATA version 18 (STATA Corp., College Station, TX, USA).

## RESULTS

### Clinical characteristics of the cohorts

During the 1-year period before e-alert implementation, 36 379 patients underwent 185 305 electrolyte measurements. Following the e-alert implementation, 37 395 patients underwent 192 378 measurements. Patient demographics, including mean age and proportion of males, as well as the proportion of inpatients and the distribution of ordering physicians’ departments, did not significantly differ between periods (standardized difference <10%) (Table 1).

**Table 1:** Demographics.

	Before e-alerts (May 2020–April 2021) (*n* = 36 379)	After e-alerts (May 2021–April 2022) (*n* = 37 395)	d (%)
Demographics			
Age, median (IQR), years	62.8 (43.2–75.7)	62.2 (43.0–75.4)	−0.4
Male, *n* (%)	17 498 (48.1)	17 805 (47.6)	−0.1
Female, *n* (%)	18 881 (51.9)	19 590 (52.4)	0.1
Number of electrolyte measurements, *n* (%)	185 305	192 378	
Inpatient	68 744 (37.1)	69 282 (36.0)	−2.3
Outpatient	116 561 (62.9)	123 096 (64.0)	2.3
Departments, *n* (%)			
Hematology	20 186 (10.9)	20 944 (10.9)	−0.02
General Surgery	15 665 (8.5)	15 641 (8.1)	−1.2
Urology	15 210 (8.2)	14 884 (7.7)	−1.7
Hepatology	12 906 (7.0)	14 112 (7.3)	1.4
Cardiology	12 785 (6.9)	11 869 (6.2)	−3.0
Nephrology	11 924 (6.4)	14 964 (7.8)	5.2
Others	96 629 (52.1)	99 964 (52.0)	−0.4

The data are shown as median (interquartile range) or *n* (%).

IQR, interquartile range; d, standardized difference.

### Nephrology referrals due to hyponatremia

Nephrology referrals due to hyponatremia increased after e-alert implementation [IRR (95% confidence interval, CI) 2.07 (1.04–4.36)]. Referrals for other electrolyte disturbances also increased (Supplementary data, Table S1 and Fig. S2).

### Changes in clinical practice and outcomes among patients with hyponatremia

Before and after e-alert implementation, 220 and 281 patients developed hyponatremia meeting e-alert criteria (serum sodium concentration <125 mEq/L). After exclusions specified in Supplementary data, Fig. S3, 166 and 235 events, respectively, were analyzed. Severity of hyponatremia was not significantly different between periods among those with and without nephrology involvement (either nephrology referral or admission to the nephrology service) (Supplementary data, Table S2). The proportion of hyponatremic events with measurements of urine sodium and osmolarity increased from 34.9% to 46.8% [odds ratio (OR) (95% CI) 1.62 (1.08–2.44)]. The proportion of urine sodium and osmolarity measurements increased irrespective of nephrology involvement. The proportion of patients who remained untreated decreased from 30.1% to 11.5% (OR (95% CI) 0.31 (0.19–0.53)]. Intravenous NaCl administration, cessation or reduction of loop or thiazide diuretics, and the use of loop diuretics (including the use for heart failure) and tolvaptan increased after implementation of e-alerts. Among hyponatremic events, 134 and 200 occurred during hospitalization before and after e-alert implementation. Median LOS decreased from 27 (15–47) days to 20 (12–36) days after e-alert implementation (*P* = .01). LOS also decreased from 7 (3–13) days to 6 (3–13) days among patients without hyponatremia (*P* = .002). With enough participants, any trivial difference eventually becomes statistically significant. For comparisons of outcome measures between groups, Cohen advocated the use of standardized effect size indices, such as the standardized mean difference (d), to quantify the magnitude of the treatment effect beyond statistical significance testing [12]. The decrease in LOS was significant for patients with hyponatremia (standardized difference −12.0%) and even greater among patients with nephrology involvement (standardized difference −59.7%). In contrast, the decrease in LOS among patients without hyponatremia was not substantial (standardized difference −8.0%) (Table 2).

**Table 2:** Measurements of urine chemistry, treatments, LOS, falls and the overcorrections of sodium before and after the initiation of e-alerts among those with hyponatremia.

	−12 to 0 months	0 to 12 months	d (%)	OR, IRR or *P*-value
The number of measurements of urine sodium concentration and osmolarity, *n*/*N* (%)^a^				
Total	58/166 (34.9)	110/235 (46.8)	24.3	1.62 (1.08–2.44)
No nephrology involvement	44/144 (30.6)	70/188 (37.2)	14.1	
Nephrology involvement^b^	14/22 (63.6)	40/47 (85.1)	50.7	
Treatments for hyponatremia, *n* (%)^c^				
Any treatment	116 (69.9)	208 (88.5)		3.19 (1.90–5.34)
Intravenous NaCl (including hypertonic NaCl)	75 (45.2)	144 (61.3)	32.7	
Hypertonic saline	11 (6.6)	9 (3.8)	−12.6	
Oral NaCl	26 (15.7)	40 (17.0)	3.7	
Cessation or reduction of loop or thiazide diuretics	10 (6.0)	28 (11.9)	20.7	
Tolvaptan	6 (3.6)	18 (7.7)	17.6	
Loop diuretic^d^	7 (4.2)	21 (8.9)	19.1	
Others	18 (10.8)	29 (12.3)	4.7	
No treatment	50 (30.1)	27 (11.5)	−47.2	0.31 (0.19–0.53)
Length of stay^e^				
Hyponatremia, median (IQR), days	27 (15–47) (*n* = 134)	20 (12–36) (*n* = 200)	−12.2	.01
No nephrology involvement, median (IQR), days	26 (15–39) (*n* = 115)	21 (12–33) (*n* = 160)	−25.0	
Nephrology involvement, median (IQR), days	31 (15–61) (*n* = 19)	20 (10–40) (*n* = 40)	−59.7	
No hyponatremia^f^	7 (3–13) (*n* = 17 968)	6 (3–13) (*n* = 18 679)	−8.0	.002
The number of falls (events per admission), *n* (%)^g^				
0	118 (88.1)	183 (91.5)		0.82 (0.44–1.50)
1	13 (9.7)	12 (6.0)		
2	3 (2.2)	4 (2.0)		
3	0	1 (0.5)		
Overcorrections of sodium levels, *n*/*N* (%)^g^	27/134 (20.1)	29/200 (14.5)	−15.0	0.64 (0.36–1.14)

^a^All patients with hyponatremia were included. If a patient experienced multiple episodes of hyponatremia, the episode with the lowest sodium concentration was selected.

^b^Either nephrology referral or admission to nephrology service.

^c^Treatments for hyponatremia were not mutually exclusive (some patients received more than one treatment).

^d^This includes the use of loop diuretics for hyponatremia associated with hyponatremia.

^e^Admissions with the lowest sodium concentration were selected for each patient.

^f^All the admissions during observation period without hyponatremia were included.

^g^Admissions with hyponatremia were included. If a patient had multiple admissions with hyponatremia, an admission with the lowest sodium concentration was selected.

d, standardized difference, standardized differences for continuous variables were calculated after log-transformation; IQR, interquartile range.

The number of falls during hospitalization was lower after e-alerts [0.14 events per admission to 0.12 events per admission; IRR 0.82 (0.44–1.50)], although this difference was not statistically significant. E-alerts were also associated with non-significant decrease in overcorrection of sodium levels (20.1% to 14.5%; OR (95% CI) 0.64 (0.36–1.14)]. After adjustment for age, sex, ambulatory versus hospitalized setting and departments, the results were not substantially different. Adjusted ORs for urine chemistry measurements was 1.66 (1.06–2.60), for treatment for hyponatremia 3.42 (2.01–5.84) and for overcorrection of serum sodium 0.58 (0.31–1.06), and the adjusted IRR for falls was 0.75 (0.36–1.57). Analyses stratified by severity of hyponatremia (serum Na ≤120 mEq/L and 121–124 mEq/L) showed that the associations of e-alert implementation with urine chemistry measurements, treatments, overcorrection and falls were similar irrespective of severity of hyponatremia (Supplementary data, Table S3).

## DISCUSSION

This is the first report of e-alerts for electrolyte disturbances. In this study, we demonstrated that e-alerts for electrolyte disturbances were not only associated with increased nephrology referrals, but also with increased measurements of urine sodium concentrations and urine osmolarity, and treatments for hyponatremia, and shorter LOS. The incidence of falls and overcorrection of serum sodium concentrations was lower after e-alerts, although the difference did not reach statistical significance.

E-alerts have been evaluated for various medical conditions, yet their clinical benefits remain controversial. E-alerts for AKI have been most intensively studied with conflicting results. An observational study reported improved AKI recognition, less progression to severe AKI, and greater recovery from AKI after e-alert implementation [2]. Conversely, a randomized controlled trial on e-alerts for AKI demonstrated that e-alerts increased nephrology referrals without changes in AKI progression, dialysis requirements or in-hospital mortality [3]. Another randomized controlled trial evaluating e-alerts combined with care bundles and educational programs reported shorter LOS [13]. E-alerts have also been evaluated in patients with heart failure. The Pragmatic trial Of Messaging to Providers about Treatment of Heart Failure [14] evaluated e-alerts recommending guideline-directed medical therapy for patients with heart failure with reduced ejection fraction. The alerts increased guideline-directed medical therapy class prescription by more than 40%. In contrast, the Risk Evaluation and its Impact on Clinical Decision-Making and Outcomes in Heart Failure trial [15] examined the impact of e-alerts on prognostic information (estimated 1-year mortality rate) of heart failure. The alerts did not affect 1-year mortality, hospitalization, the placement of defibrillators or referral to palliative care. Similarly, e-alerts for thromboprophylaxis did not increase the use of thromboprophylaxis during hospitalization for acute illness in a randomized controlled trial [16]. Many e-alerts were ignored by treating physicians and thromboprophylaxis use in the control group was higher than expected. These mixed findings suggest that effective e-alerts should target the condition that are unrecognized, treatable or linked to actionable interventions, and associated with outcomes that treatment can improve.

Electrolyte disturbances, particularly hyponatremia, may satisfy these conditions. First, hyponatremia is often under-recognized and mismanaged [9]. The measurements of urine sodium and osmolarity were performed only in 13.6% [17], 28.8% [18] or 65.5% [19] among hospitalized patients with serum sodium <135, <128 or <125 mEq/L, respectively. Patients with hyponatremia did not receive treatment in 13.7% [19], 32.6% [20] or 30% [17] for serum sodium concentration <125, <130 or <135 mEq/L, respectively. Second, hyponatremia is treatable. Water intoxication can be treated with free water restriction, sodium depletion with intravenous or oral NaCl, SIAD with water restriction, oral NaCl or vaptans [21]. Third, treating hyponatremia might improve outcomes. Correction of hyponatremia has been associated with increased procollagen type 1 N-terminal propeptide, a marker of osteoblast function [6]. In a randomized controlled trial comparing empagliflozin to placebo for patients with SIAD, empagliflozin improved both serum sodium concentration and neurocognitive function [7]. Hyponatremia was associated with gait disturbance and correction of hyponatremia improved this disturbance [8]. Among patients with profound hyponatremia, treatment was associated with lower mortality than no treatment [hazard ratio (95% CI) 0.37 (0.17–0.78)] [9]. Despite potential benefits, few studies examined e-alerts for hyponatremia. One study implemented e-alerts for patients at high risk of hyponatremia and demonstrated that the incidence of hyponatremia decreased from 29% to 14% [22]. Another study examined the e-alert for hyponatremia (sodium concentration ≤116 mEq/L) and showed that approximately 40% of cases were due to adverse drug reactions [23]. No studies have investigated the outcomes after e-alert implementation. In the present study, urine electrolyte and osmolarity testing among patients with hyponatremia increased after e-alert implementation, regardless of nephrology involvement. This suggests improved physician recognition and evaluation of hyponatremia. Treatments for hyponatremia also increased. These changes in clinical practice might have contributed to improvement in outcomes including shorter LOS. The findings were compatible with a randomized, cross-over trial showing that LOS was shorter by 4 days if the patients with hyponatremia were diagnosed and treated early [24]. In this randomized trial, the incidence of falls was also significantly lower among early treatment group, although the impact of e-alert on falls was not statistically significant in our study.

The strength of this study is that it is the first report to evaluate the impact of e-alerts for electrolyte disturbances. It involved a large cohort of more than 70 000 patients with over 370 000 electrolyte measurements. The e-alert system covered multiple electrolyte abnormalities (sodium, potassium, calcium and magnesium) and demonstrated associations not only with an increase in nephrology referrals but also measurable improvements in clinical practice, including increased rates of urine sodium and osmolality testing, and shorter hospital stays. Furthermore, the intervention was implemented as a part of routine electronic medical record system, suggesting that such alerts can be feasibly integrated into existing hospital workflows and may be readily generalizable to other institutions.

This study has several limitations. First, it was a single-center retrospective observational study comparing before and after the initiation of e-alerts. The lack of control group precluded the establishment of causal relationships between the e-alert implementation and observed changes. Multicenter studies using cluster randomization or stepped-wedge randomization design could more robustly evaluate the effects of e-alerts for electrolyte disturbances. Second, detailed physician responses to alerts were unavailable. Although urine chemistry measurements increased after e-alert implementation, these tests may not be required in every hyponatremic patient, for example in clear hypervolemic presentations. Increased measurements may not always improve clinical practice. Similarly, not all cases of hyponatremia require nephrology referrals (for example, hyponatremia associated with heart failure). Although we described an increase in the number of nephrology referrals after e-alerts, it was difficult to ascertain the proportion of referrals that were truly necessary and beneficial. Third, mild or moderate electrolyte abnormalities were not included in the alert system, which may limit its applicability to broader clinical settings. Fourth, we did not assess patient-centered outcomes such as readmission rates, which would be important to evaluate in future multicenter prospective studies. Fifth, analyses were not adjusted for multiple comparisons.

In conclusion, the implementation of e-alerts for electrolyte disturbances was associated with increased nephrology referrals, urinary sodium and osmolarity measurements, and treatments of hyponatremia, and shorter LOS.

## Supplementary Material

sfag383_Supplemental_Files

## Data Availability

The data are available upon reasonable requests to the corresponding author.
