# Supplementary material for: Changes in clinical practice patterns and outcomes of hyponatremia after implementation of electronic alerts for electrolyte disturbances
Source: Clin Kidney J. 2026 Oct 30;19(11):sfag383. doi: 10.1093/ckj/sfag383 (PMC13760206; doi:10.1093/ckj/sfag383)
Supplement: Supplementary file 1 [file sfag383_supplemental_files.zip › Supplementary Table revised 20260908.docx]

Supplementary Table 1. Number of nephrology referrals for electrolyte disturbances

| Months | Referrals  for electrolyte disturbances | IRR  （95% CI） |
| --- | --- | --- |
| -12 to -6 | 28 | 1 (ref) |
| -6 to 0 | 34 |  |
| 0 to 6 | 50 | 1.68 (1.22-2.33) |
| 6 to12 | 58 |  |

IRR: incidence rate ratio

Incidence rate was calculated as the number of referrals per electrolyte measurements.

**Supplementary Table 2. Severity of hyponatremia (lowest sodium concentration) for those with and without nephrology referrals**

|  | Before e-alerts  (mEq/L) | After e-alerts  (mEq/L) | p (comparison of before and after e-alerts) |
| --- | --- | --- | --- |
| Nephrology involvement | 120.5 (119-122) | 121 (118-122) | 0.82 |
| No nephrology involvement | 122 (120-124) | 122 (119-124) | 0.86 |
| p (comparison of with and without nephrology referrals) | 0.08 | 0.06 | NA |

P values were by Man-Whiteny U test.

**Supplementary Table 3. Measurements of urine electrolytes and osmolarity and length of stay among patients with hyponatremia stratified by severity of hyponatremia**

|  | Before | After | d | OR | P for interaction |
| --- | --- | --- | --- | --- | --- |
| Measurements of urine electrolytes and osmolarity | | | |  |  |
| Na 121-124 mEq/L | 31/111 (28.2) | 59/150 (39.3) | 24.3 | 1.56 (0.78-3.08) | 0.89 |
| Na≤120 mEq/L | 27/55 (49.1) | 51/85 (60.0) | 22.0 | 1.65 (0.97-2.80) |  |
| Length of stay (days) | | | |  |  |
| Na 121-124 mEq/L | 29.5 (16.5-47)  (n=84) | 22 (13-39)  (n=123) | -12.1 | P=0.03 |  |
| Na≤120 mEq/L | 21 (14-37)  (n=50) | 18 (10-28)  (n=77) | -11.9 | P=0.16 |  |
| Fall (incidence rate ratio) | | | |  |  |
| Na 121-124 mEq/L | 1 (ref) | 1.13 (0.47-2.72) | NA |  | 0.20 |
| Na≤120 mEq/L | 1 (ref) | 0.41 (0.11-1.53) |  |  |  |
| Overcorrection | | | |  |  |
| Na 121-124 mEq/L | 12/84 (14.3) | 9/123 (7.3) | -22.6 | 0.44 (0.18-1.11) | 0.33 |
| Na≤120 mEq/L | 15/50 (30.0) | 20/77 (25.8) | -9.0 | 0.81 (0.36-1.79) |  |

d: standardized difference
